# Effects of silencing epididymal vascular endothelial growth factor (VEGF) expression on hyaluronidase (HYD) activity in arsenic poisoning rats through downregulating VEGF receptor 2 (VEGFR2)

**DOI:** 10.1080/21655979.2021.1915726

**Published:** 2021-04-27

**Authors:** Yan-ping Dai, Xiao-qin Gao

**Affiliations:** a Department of Pathology and Pathophysiology, College of Basic Medical Science, Guizhou Medical University, Guiyang, Guizhou, China; b Department of Internal Medicine, People’s Hospital in Yueyanglou District, Yueyang, Hunan, China; c Centre for Reproductive Research, National School of Medicine Guiyang Medical University Magic, Guiyang, Guizhou, China; d Department of Histology and Embryology, Zunyi Medical and Pharmaceutical College, Zunyi, Guizhou, China

**Keywords:** Vascular endothelial growth factor (VEGF), arsenic poisoning, hyaluronidase (HYD) activity, RNA interference, VEGF receptor 2 (VEGFR2)

## Abstract

RNA interference (RNAi) was used to investigate the role of epididymal vascular endothelial growth factor (VEGF) gene expression on sperm hyaluronidase (HYD) in a rat model of arsenic poisoning and to identify a new gene therapy target for male infertility caused by arsenic poisoning. The Rat model of chronic arsenic poisoning was established. And we found that positive expression of VEGF and VEGF receptor 2 (VEGFR2) was observed by Immunohistochemical staining in the epididymal tissues of arsenic-exposed rats. Subsequently, VEGF-shRNA-1, VEGF-shRNA-2 and VEGF shRNA-3 expression vectors containing epididymal VEGF-shRNA lentivirus were constructed and injected into the bilateral epididymis of each group of rats (Control group, NC-shRNA negative infection group, VEGF-shRNA-1 group, VEGF-shRNA-2 group, VEGF-shRNA-3 group) (n = 10 per group). Compared with the negative infection group and the normal control group, the expression of VEGF and VEGFR2 mRNA and protein levels were significantly decreased following epididymal infection. In addition, the HYD activity was all significantly lower than that in the normal control group and the negative infection group. Taken together, epididymal VEGF gene silencing may inhibit the activity of sperm HYD through downregulating VEGFR2.

## Graphical abstract

**Figure:**
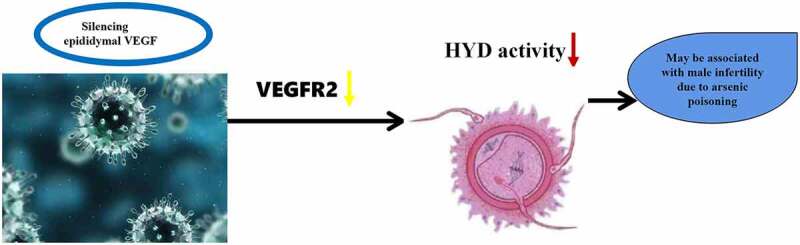

## 1. Introduction

Chronic arsenic poisoning can cause damage to the reproductive system. There are estimated to be more than 3 million people suffer from chronic arsenic in China [1]. Chronic exposure to high-arsenic water may cause damage to the chromosomes of eukaryotic cells and affect their progeny [2]. Studies have proved that chronic exposure to arsenic can affect reproduction and development, highlighting the urgent need to clarify the mechanism of its reproductive toxicity [3,4].

The epididymis is the site at which mammalian sperm mature, become motile and acquire the ability to fertilize. The epididymis can also store and protect sperm. By passing through the epididymis, sperm constantly interact with proteins that are specifically expressed in the microenvironment of the seminiferous tubule lumen. It is during this passage that sperm reacts with the zona pellucida and fertilize. The acquisition of these key properties depend mainly on the participation of proteins synthesized and secreted specifically by the epididymis [5].

Vascular endothelial growth factor (VEGF) is related to angiogenesis, and participates in the division and proliferation of endothelial cells. VEGF receptor 2 (VEGFR-2) is one of the receptors for VEGF, increase vascular permeability and can promote the growth and metastasis of tumors [6]. The combination of VEGF/VEGFR2 can inhibit apoptosis, promote endothelial cell proliferation, and improve vascular permeability [7–9]. Studies have also proved that VEGF and its receptor 2 were expressed in the epididymis, indicating that it may be may be involved in male reproduction [10–12].

RNAi interference technology is a new target gene suppression technology with high efficiency, specificity, stability and safety. Furthermore, anti-tumor angiogenesis gene therapy is a particularly popular are of research in tumor therapy at present [13,14]. VEGF is the primary factor affects survival of vascular endothelial cells. There have been several extensive studies attempting to inhibit the angiogenesis of tumors by targeting VEGF/VEGFR2. Indeed, blocking the VEGF/VEGFR2 signal transduction pathway by high potency gene suppressor-RNA interference technology has become a particular hotspot in anti-tumor gene therapy. VEGF is associated with tumor progression [15]. However, little has been reported about the role of VEGF in male reproduction the role of VEGF in the male reproduction system.

With the deepening of research, semen enzymology examination has attracted more and more attention. Hyaluronidase (HYD) is a key enzyme in the process of fertilization. The activity of this enzyme can be used as a functional index to judge the fertility of men [16]. Previous research results of our group found that male infertility was related to germ cell apoptosis and key enzymes in acrosome [17,18]. Therefore, it is necessary to explore the mechanism and correlation of male infertility caused by chronic arsenic poisoning from the perspective of enzymology. At present, there are few reports on whether interference with VEGF gene in rat epididymis can affect the activity of HYD, a key enzyme during fertilization.

In this study, we aimed to observe the expression of VEGF and VEGFR2 in the epididymis of rats exposed to arsenic. The effect of VEGF on sperm HYD activity was further investigated by interfering with its expression to elucidate the molecular mechanism of arsenic poisoning on rat reproduction.

## 2. Materials and methods

### 2.1. Animals and grouping

A total of 40 SPF grade male Sprague-Dawley (SD) rats (approximately 18 months old) were purchased from the Guizhou Medical University Laboratory Animal Center. This experimental scheme was approved by the Animal Care Welfare Committee of guizhou medical university (Animal license: SCK (Guizhou), 2018–0001). All applicable international, national, and institutional guidelines for the care and use of animals were followed.

The sodium arsenite was obtained from Beijing Chemical Factory (China). The rats were housed singly per cage under controlled condition of ambient temperature (20–25℃), humidity (60–67%), and photoperiod controlled room (light/dark: 12 h/12 h). After adaptive feed for one week, according to previous findings [19] in this research, the rats were divided equally into 4 groups, respectively, for the high-dose (60.0 mg/L sodium arsenite in water), middle-dose (12.0 mg/L sodium arsenite in water), and low-dose (2.4 mg/L sodium arsenite in water) dose arsenic infected group and the control group (distilled water), with 10 animals per group. Then treated with the arsenism through drinking water for 6 consecutive months.

### 2.2. Immunofluorescence staining (IF)

Thin sections of epididymis (5 μm) were rehydrated through a graded series of ethanol to PBS. Hydrogen peroxide was then added to block endogenous peroxidase. Sections were then blocked with goat serum at room temperature for 30 min prior to overnight incubation with VEGF and VEGFR2 antibody (1:1000 dilution, GeneTex, USA) at 4℃. The next morning, sections were allowed to recover for 40 min at room temperature before being incubated with FITC-labeled goat anti rabbit IgG (1:500) for 2 h at 37℃. The samples were sealed with glycerin-sodium bicarbonate and observed by fluorescent microscopy. Images were acquired using a fluorescent microscope (OlympusBX51). Data represent the means of three to five independent trials.

### 2.3. Immunohistochemical staining (IHC)

The sections of epididymis were dewaxed and rehydrated. Citrate buffer was added to the tissues for 30 min for antigen retrieval. 3% hydrogen peroxide was added to the sections for 5 min then washed in Tris-buffered saline(TBS). TBS containing 5% bovine serum albumin was added to the sections, then incubated with rabbit polyclonal antibody (VEGF and VEGFR2) at 4℃. After three TBS washes, the appropriate secondary antibody conjugated to biotin goat anti-rabbit was added to the slides for 30 min, at 37℃ diluted 1:500 in the blocking mixture. DAB (ZSGB-BIO, China) was then used for developing for 5 min. The pictures were analyzed by Image J analysis software.

### 2.4. Construction of VEGF-shRNA lentiviral vector

The lentiviral packaging plasmids, pCMV-dR8.9 and PCMV-Type-G were obtained from Addgene (USA). We used the epididymal-specific VEGF gene sequence (NCBI accession number: nm_031836.3) reported by gene bank and RNAi targeting design principles to design three interference targets for VEGF along with a negative control. The primers were synthesized by Shanghai Bioengineering co., LTD (Shanghai, China). Three VEGF shRNA lentiviral vectors were constructed with reference to existing research methods [20,21]. The three target shRNA sequences were 5ʹ-GCAGCTATTGCCGTCCAATTGCTCGAGCAATTGGACGGCAATAGCTGCTTTTTTG-3ʹ for shRNA-1, 5ʹ-CCGGGCGGATCAAACCTCACCAAAGCTCGAGCTTTGGTGAGGTTTGATCCGCTTTTTTG-3ʹ for shRNA-2, and 5ʹ-CCGGGCCAGCACATAGGAGAGATGACTCGAGTCATCTCTCCTATGTGCTGGCTTTTTTG-3ʹ for shRNA-3.

### 2.5. Infection of rat epididymal tissues with lentiviral vectors

Fifty healthy male SD rats were anesthetized with chloral hydrate and sterilized with surgical towels. We then exposed the skin of the epididymis and carefully dissected and separated the epididymis; 200 μl of recombinant and negative control lentiviruses were injected into the bilateral epididymis of each group of rats (control group, NC-shRNA negative infection group, PL-VEGF-shRNA-1 infection group, PL-VEGF-shRNA-2 infection group, PL-VEGF-shRNA-3 infection group; n = 10 per group). After 14 days of feeding in a sterile animal room, the rats were anesthetized, and both epididymal tissues were dissected under sterile conditions for follow-up experiments.

### 2.6. Quantitative real-time PCR (qRT-PCR)

Total RNA was extracted from the epididymal tissues of each group. Then the RNA was reverse transcribed into cDNA using QuantiTect Reverse Transcription Kit (QIAGEN) according to the manufacturer’s protocol. PCR reaction conditions: First denaturation at 95℃ for 3 min, then 95℃ for 7 s, annealing at 57℃ for 10 s, then extension at 72℃ for 15 s (40 cycles). The relative expressions of VEGF and VEGFR2 were then calculated using the 2^－ΔΔCt^ method. The primers used for qRT-PCR were shown in Table 1.

**Table 1.** Primer sequences

Gene	Sequence
GAPDH	Forward: 5′-CAAGTTCAACGGCACAGTCAA-3′
	Reverse: 5′-CGCCAGTAGACTCCACGACA-3′
VEGF	Forward: 5′-GAGAACGTCACTATGCAGATC-3′
	Reverse: 5′-TTTCTCCGCTCTGAACAAGG-3′
VEGFR2	Forward: 5′-TCATTATCCTCGTCGGCACT-3′
	Reverse: 5′- TAAGGCAAGCGTTCACAGC-3′

### 2.7. Western blotting

Total protein was extracted from each epididymal tissue. Proteins (20 μg/sample) were separated by 12% SDS-PAGE gel and then transferred to the PVDF membrane. After blocking with 5% nonfat milk at 37℃ for 2 h, the membranes were incubated with VEGF antibody (1:1000 dilution) and VEGFR2 antibody (1:1000 dilution) at 4℃ overnight. The membrane was washed three times with Tris buffered saline (TBST) and then incubated with HRP-labeled goat-anti-rabbit IgG at 37℃ for 2 h. The membrane was then washed three times in TBST (10 mins each time). An ECL chemiluminescence kit was then used to detect positive antibody binding on the membrane. Banding patterns were then analyzed using a gel image analysis system (NIH).

### 2.8. Detection of HYD activity

The modified sodium hyaluronate-gelatin substrate membrane method was used to make and fix the substrate membrane [22]. The spermatozoa suspension that had been washed, centrifuged and diluted was coated on the prepared substrate membrane, evenly spread, and incubated in a constant temperature and humidity box at 37℃ for 2 h, then observed and stained. After termination of incubation, the sections were stained with appropriately diluted Jincheng blue ink, washed with running water, dried naturally for 3 h, and the HYD activity was observed thoroughly with light microscope. (3 sections were randomly selected from each group, each of which was randomly observed in 10 fields to observe 200 sperm heads. The sperm was dyed dark blue and the bright halo ring around the sperm head was considered as positive reaction, and the microscale was used to measure. The diameter of the bright ring indicated the enzyme activity, and the larger the halo diameter, the higher the brightness indicated the better the activity).

### 2.9. Statistical analysis

Statistical Product and Service Solutions (SPSS) 13.0 software was used to process all statistical data. Data are expressed as mean ± standard deviation (SD). One-way analysis of variance (ANOVA) was used for comparison between different groups. The LSD test was used for the comparison of two groups. *P* < 0.05 was considered to be statistically significant.

## 3. Results

### 3.1. Positive expression of VEGF and VEGFR2 on epididymal tissues of rats

**Figure 1.**
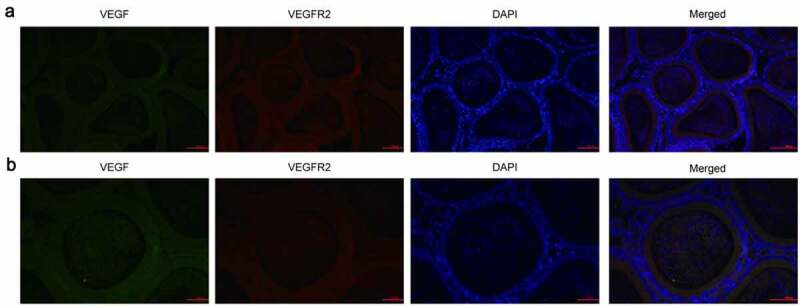
Expression of VEGF and VEGFR2 was detected by immunofluorescence. (a): Magnification, × 200, (b): Magnification, × 400. Immunofluorescence staining showed that cells positive for VEGF and VEGFR2 were predominantly observed in the cytoplasm of all principle cells, some of the interstitial small vascular endothelial cells, and in some blood capillaries and sperm in the ductus epididymitis. VEGF, Vascular endothelial growth factor; VEGFR2, VEGF receptor 2

We first collected epididymal tissues from rats and IF detected the expression of VEGF and VEGFR2. VEGF positive reactivity (green fluorescence) was observed in the principle cells of the entire epididymal epithelium, as well as in the cilia near the free surface of the lumen and in some sperm in the ductus epididymidis. VEGFR2 (red fluorescence) was predominantly observed in the cytoplasm of principle cells, the small vascular endothelial cells in interstitial, some blood capillaries and sperm in the ductus epididymidis. However, no expression of VEGF and VEGFR2 was detected on clear cells, halo cells and basal cells (Figure 1(a,b)).

### 3.2. VEGF and VEGFR2 were downregulated in epididymal tissues of arsenic-exposed rats

**Figure 2.**
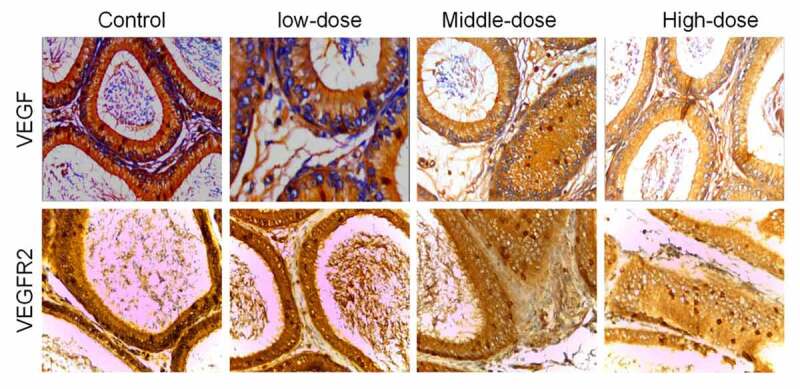
VEGF and VEGFR2 were downregulated in epididymal tissues of arsenic-exposed rats. Representative images of immunohistochemical staining using antibodies against VEGF (a) and VEGFR2 (b). VEGF, Vascular endothelial growth factor; VEGFR2, VEGF receptor 2

IHC was further used to detect the expression of VEGF and VEGFR2 in arsenic-exposed rats. Compared with the control group, the average optical density of VEGF and VEGFR2 in each sodium arsenite exposure groups were significantly decreased (*P* < 0.05). And with the increase of sodium arsenite concentrations, the average optical density of VEGF and VEGFR2 were decreased (Figure 2 and Table 2).

**Table 2.** Mean expression levels of VEGF and VEGFR2 in the epididymis of rats, as determined by immunohistochemical analysis (n = 10, Mean *±* SD)

Group	VEGF	VEGFR2
Normal control group	0.28 ± 0.03	0.24 ± 0.02
Low dose arsenic infected group	0.26 ± 0.05*	0.08 ± 0.01*
Middle dose arsenic infected group	0.23 ± 0.04*	0.06 ± 0.01*
High dose arsenic infected group	0.22 ± 0.17*	0.05 ± 0.01*

*compared with control group, *P* < 0.05.

### 3.3. mRNA expression of VEGF and VEGFR was decreased by shRNA lentiviral vectors

To determine whether low VEGF expression is consistent with the effects of arsenic exposure in rats, we constructed three VEGF shRNA lentiviral vectors. VEGF-sh-RNA-1, 2, 3 lentiviral vectors were injected into the bilateral epididymis of rats, and epididymal tissues were subsequently collected from each group of rats. The expression of VEGF and VEGFR2 mRNA and protein in the tissues were then detected by QPCR and WB, respectively.

**Figure 3.**
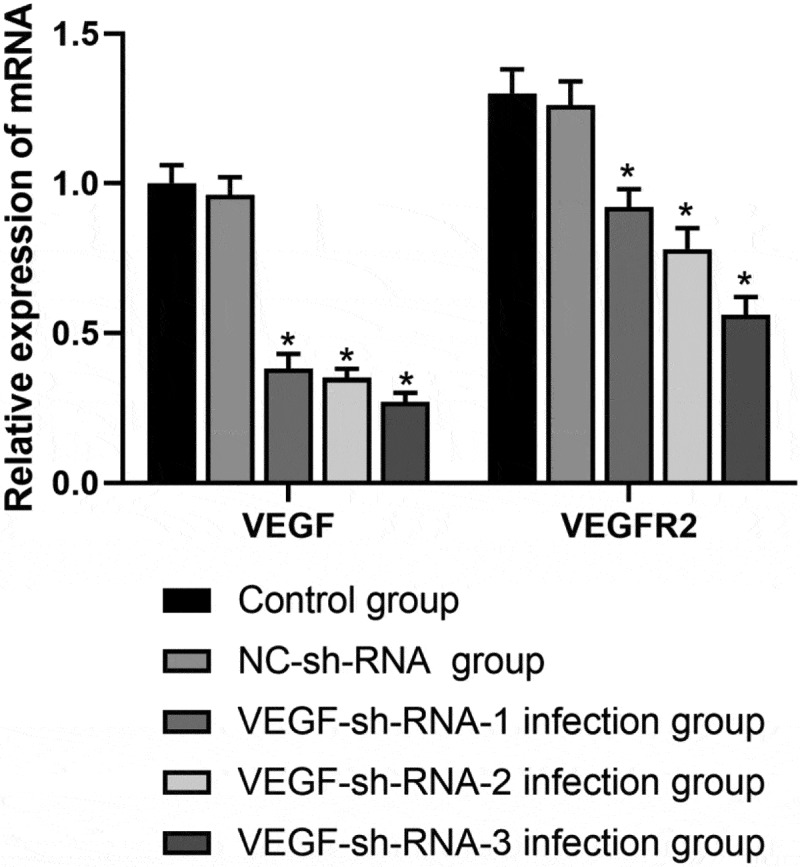
mRNA expression levels of VEGF and VEGFR2 in the epididymis of rats after transfection with VEGF-shRNA. Real-time PCR was used to measure the expression levels of VEGF and VEGFR2 in the epididymis of rats in different treatment groups following transfection with VEGF-shRNA. Data are represented as the mean ± SD. *P < 0.05 vs. control group. VEGF, Vascular endothelial growth factor; VEGFR2, VEGF receptor 2

AS shown in Figure 3, the mRNA expression levels of VEGF and VEGFR2 in the VEGF-sh-RNA-1, 2, 3 infected groups were significantly decreased than those in the control group and the NC-shRNA group (*P* < 0.05), especially in the VEGF-shRNA-3 group.

**Figure 4.**
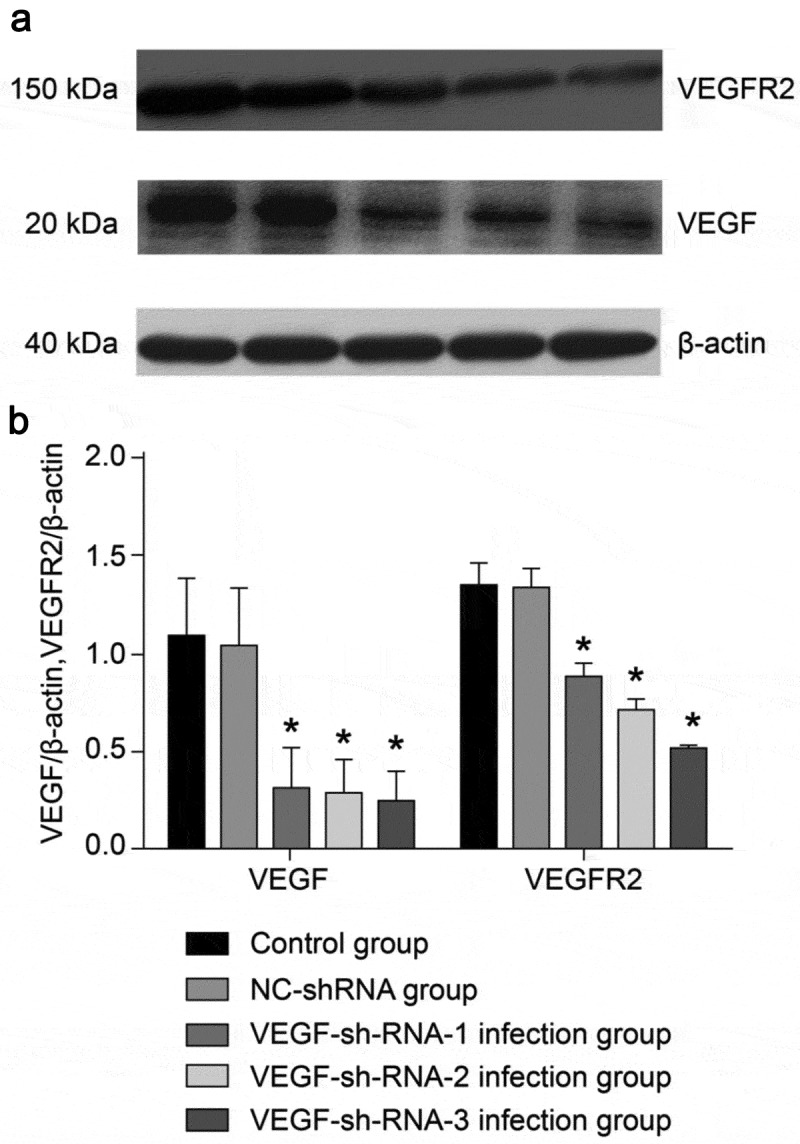
Protein expression levels of VEGF and VEGFR2 in the epididymis of rats after transfection with VEGF-shRNA. (a) The protein expression of VEGF and VEGFR was measured by Western blot. (b) Quantification analysis of the protein expression based on immunoblotting results. Data are shown as the mean ± SD (n = 3) and were analyzed by one-way analysis of variance (ANOVA). *P < 0.05 vs. control group. VEGF, Vascular endothelial growth factor; VEGFR2, VEGF receptor 2

The expression of VEGF and VEGFR2 proteins in the epididymal tissue of rats from each group were shown in Figure 4. After recombinant lentivirus had infected the epididymal tissue of rats, we found no significant difference between the NC-sh-RNA negative group and the control group (*P* > 0.05). However, the protein expression levels of VEGF and VEGFR2 in the VEGF-shRNA-1, 2, 3 infection groups were significantly lower than that of the normal control group and the NC-shRNA negative infection group (*P* < 0.05).

### 3.4 VEGF-shRNA decreased sperm hyaluronidase activity in epididymis of rat

**Figure 5.**
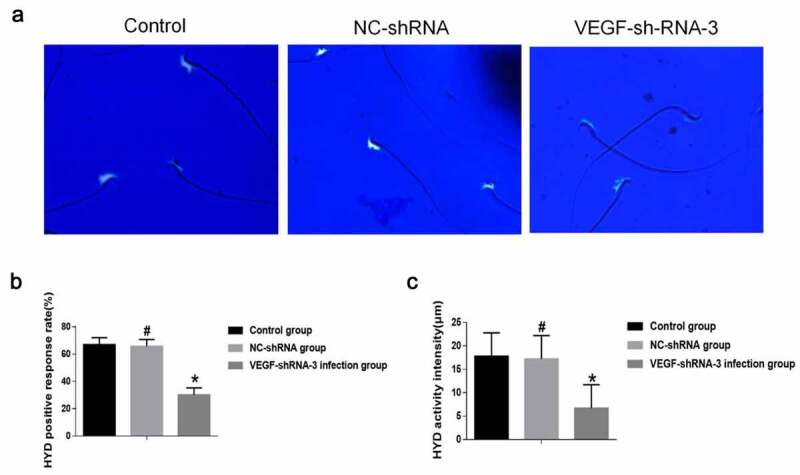
VEGF-shRNA decreased sperm hyaluronidase (HYD) activity in epididymis of rat. (a) The positive expression of HYD was detected by Fluorescent staining. Magnification, × 400., (b) The HYD positive response rate. (c) The HYD activity was measured by the mature substrate membrane method. *p < 0. 05 vs. control group. ^#^P < 0.05 vs. VEGF-shRNA-3 group

HYD is highly biologically active, present in high concentrations in the testis and localized to the head and acrosome of spermatozoa [23]. Microscopic results of HYD activity after 3 h incubation and staining of sperm in rat caudal epididymis were observed. Fluorescent staining results showed positive expression of HYD in the sperm head (Figure 5(a)). In addition, the HYD positive rate and reaction intensity of epididymal sperm in the VEGF shRNA-3 infection group were decreased then that in the control group and the NC-shRNA-negative infection group (*P* < 0.05). The HYD positive rate and reaction intensity of epididymal sperm was no significant difference between the control group and the NC-shRNA negative infection group (*P* > 0.05) (Figure 5(b,c)).

## 4. Discussion

Chronic exposure to arsenic showed a direct action of suppression on the spermatogenesis and maturation, and even lead to infertility [24,25]. It is known that abnormalities in epididymal function can affect sperm maturation, thus leading to male infertility. A series of programmed processes, such as the formation of sperm motility, the improvement of acrosome function, along with sperm and egg recognition and fusion, are dependent on the epididymal microenvironment [26]. In recent years, studies have shown that VEGF is related to reproduction [27]. VEGF and VEGFR2 are known to be expressed in semen, participate in the development and maturation of sperm and have been associated with the fertilization ability of sperm [28,29]. It also has been reported that VEGF can regulate male reproduction by acting on its receptors [27], thus directly affecting male fertility [28]. Herein, we speculate that VEGF may be correlated with male infertility caused by chronic arsenic poisoning.

Our studies showed that VEGF and VEGFR2 were mainly observed in the cytoplasm of principal cells, small interstitial endothelium, capillaries and sperm in the ductus epididymidis. In addition, we found that the expression of VEGF and VEGFR2 decreased with the increase of arsenic poisoning dose, which may be that arsenic damage to the vascular permeability of rat epididymis, thus affecting the expression of VEGF and VEGFR2, indicating it may relate with infertility. These results revealed that arsenic poisoning increased the expression of VEGF and VEGFR in rat epididymal tissues. Korpelainen et al. reported that the overexpressions of VEGF in the testis and epididymis of rats could cause infertility in transgenic mice [30]. These studies suggest that high expression of VEGF can be involved in reproductive disorders in rats.

Further we successfully constructed a VEGF-shRNA recombinant expression lentiviral plasmid. After injection of rat epididymal tissues, RT-PCR and western blotting results showed that the mRNA and protein expression of VEGF and VEGFR in epididymal tissues of the NC-shRNA −1, 2 and 3 infection groups were significantly lower than those in the control group and the NC-shRNA negative infection group. We speculated that the VEGFR2 was induced by VEGF and thus affects male reproduction.

HYD exists in the sperm acrosome and is a key enzyme in the fertilization process [31]. The activity of HYD can be used as a functional indicator to judge the fertility of male. Previous study found that male infertility was correlated with germ cell apoptosis and acrosomal HYD [32]. The epididymis is the site of sperm maturation. HYD in sperm acrosome is a key enzyme in the process of fertilization, and the positive rate of HYD and the reduced activity of the enzyme are important factors leading to male infertility [33,34]. Researchers have found that reduced semen HYD activity may cause infertility [22]. Therefore, the mature substrate membrane method of our research group can not only detect HYD activity in the sperm acrosome of rats exposed to chronic arsenic, but also estimate the strength of HYD activity in a single sperm [35]. The results of this study showed that the positive rate and activity intensity of HYD in the VEGF-shRNA infected group were significantly decreased compared with the control group. We speculate that the reduced activity of HYD will lead to the reduced ability of sperm to dissolve the extracellular matrix of the cumulus, which is not conducive to the passage of sperm through the cumulus, resulting in the weakened ability of sperm to act with the egg cell is reduced, impeding fertilization, affecting fertility, and ultimately leading to the low success rate of in vitro fertilization.

## 5. Conclusion

In conclusion, we found that arsenic poisoning increased the expression of VEGF and VEGFR in rat epididymis, while interference with VEGF downregulated VEGFR2 and thus decreased HYD activity. It is suggested that VEGF may act as a new indicator for the diagnosis and treatment of infertility due to arsenic poisoning. However, it remains to be elucidated in more details in future investigation.

## Supplementary Material

Supplemental Material

## Highlight

1. Positive expression of VEGF and VEGFR2 was observed in the epididymal tissues of arsenic-exposed rats.
2. The expression of VEGF and VEGFR2 were significantly decreased by shRNA lentiviral vectors.
3. Interfering with VEGF significantly inhibited hyaluronidase activity in epididymal tissue.
