# Supplementary figures and images for: Effects of silencing epididymal vascular endothelial growth factor (VEGF) expression on hyaluronidase (HYD) activity in arsenic poisoning rats through downregulating VEGF receptor 2 (VEGFR2)
Source: Bioengineered. 2021 Apr 27;12(1):1351–9. doi: 10.1080/21655979.2021.1915726 (PMC8806240; doi:10.1080/21655979.2021.1915726)

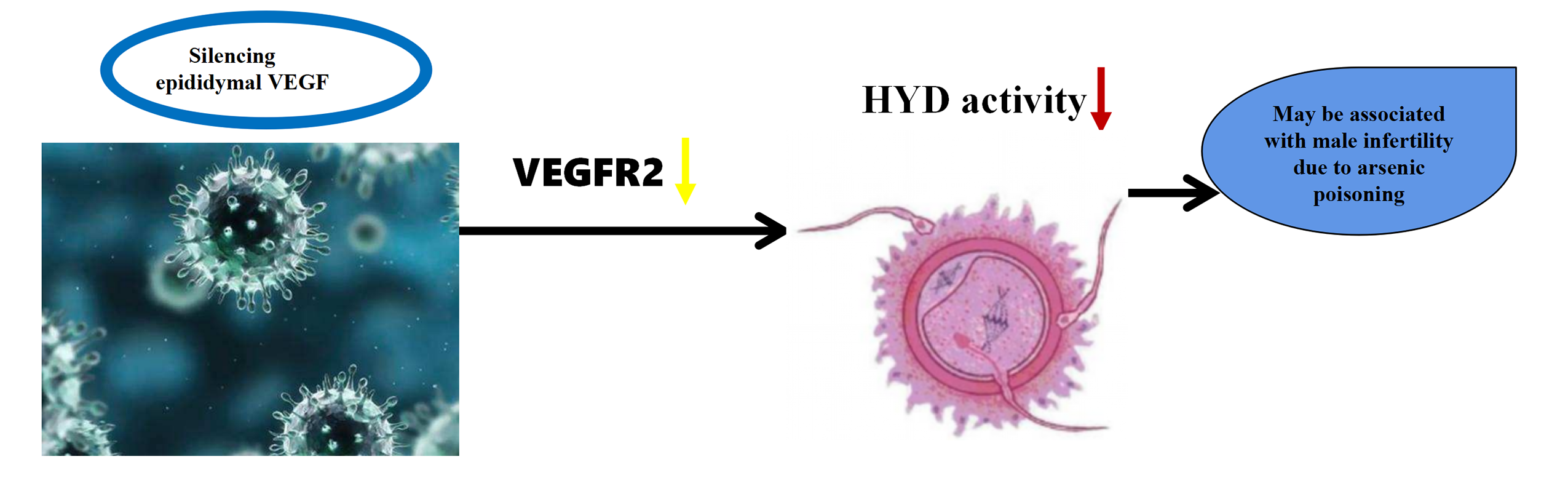

Supplement: Supplemental Material [file KBIE_A_1915726_SM6626.tif]
